# Risk factors for lymph node metastasis and surgical methods in patients with early-stage peripheral lung adenocarcinoma presenting as ground glass opacity

**DOI:** 10.1186/s13019-020-01167-2

**Published:** 2020-08-12

**Authors:** Yongming Wang, Lijun Jing, Gongchao Wang

**Affiliations:** 1 Department of Thoracic Surgery, Shandong Provincial Hospital, Cheeloo College of Medicine, Shandong University, Jinan, 250021 Shandong China; 2 Department of Thoracic Surgery, Weifang NO.2 People’s Hospital, Yuanxiao Street, Kuiwen District, Weifang, 261041 China; 3 Department of Anesthesiology, Weifang NO.2 People’s Hospital, Yuanxiao Street, Kuiwen District, Weifang, 261041 China

**Keywords:** Lung adenocarcinoma, Ground glass nodule, Lymph node metastasis

## Abstract

**Background:**

It is difficult to predict lymph node metastasis in patients with early lung cancer. Pure ground glass opacity (GGO) on computed tomography indicates an early-stage adenocarcinoma that can be removed by limited resection or lobectomy without the need for mediastinal lymph node dissection or sampling, and lung adenocarcinoma with GGO therefore has a good prognosis. We examined the incidence and risk factors of lymph node metastasis in patients with clinical stage IA lung adenocarcinoma.

**Methods:**

We retrospectively analyzed clinical data for 327 patients with stage IA peripheral lung cancer treated in our hospital from March 2014 to December 2018. The patients were divided into four groups according to computed tomography signs. Lobectomy and systematic lymph node dissection were performed in all patients. Correlations between lymph node metastasis and clinical pathological factors were analyzed by logistic regression.

**Results:**

Among the 327 patients, 26 (7.95%) had lymph node metastasis. No patients with pure GGO or GGO-dominant types had lymph node metastasis. Logistic regression identified tumor diameter, solid content, plasma carcinoembryonic antigen (CEA) level, pathological type, lymphovascular invasion, and pleural invasion as factors related to the presence of lymph node metastasis.

**Conclusions:**

Tumor diameter, solid component ratio, plasma CEA level, pathological type, vascular tumor thrombus, and pleural invasion are possible independent risk factors for lymph node metastasis in patients with stage IA lung adenocarcinoma. In contrast, lymph node metastasis is rare in patients with pure GGO or GGO-dominant lung adenocarcinoma.

## Background

Lung cancer is the main cause of cancer-related death [1]. However, although the 5-year survival rate of lung cancer is only 15%, this can be increased to about 50% by the timely diagnosis and treatment of early-stage lung cancer [2]. Lung adenocarcinoma is the most common type of lung cancer, accounting for more than 50% of non-small cell lung cancers, and the percentage is continuing to increase [3].

Progress in computed tomography (CT) has led to an increase in the detection rate of early lung nodules, including many early lung cancers [4], with relatively low malignancy and a good postoperative prognosis. Lung nodules can be divided into the following categories according to the ground glass opacity (GGO) findings: pure GGO (PGGO), mixed GGO (MGGO) or partial-solid GGO, and solid GGO (SGGO).

Compared with the seventh edition, the eighth edition of the TNM staging of lung cancer divided T1a into T1a and T1b, and changed the original T1b to the new T1c as follows: T1a (≤ 1 cm), T1b (> 1 to ≤2 cm), and T1c (> 2 to ≤3 cm) [5]. In 2011, the International Association for Lung Cancer Research, the American Thoracic Society, and the European Respiratory Society jointly issued a new international multi-disciplinary classification standard for lung cancer [6]. The incidence of lymph node metastasis in patients with lung cancer with GGO is currently unclear, and the need for lymph node cleaning during surgery is controversial. In this study, we retrospectively examined the incidence and risk factors of lymph node metastasis in patients with clinical stage IA peripheral lung adenocarcinoma with GGO findings, with the aim of providing evidence for future clinical applications.

## Methods

We retrospectively analyzed the clinical data for 516 patients who underwent surgical resection at the Department of Thoracic Surgery, Weifang NO.2 People’s Hospital, shandong, China, from March 2014 to December 2018.

All patients underwent routine pulmonary function examination, craniocerebral magnetic resonance imaging or CT, systematic bone scan, abdominal ultrasound or CT to exclude possible metastases, and analysis of related tumor markers. After plain or enhanced CT scans, all images were transferred to an AW4.6 workstation (GE Healthcare, Chicago, IL, USA) for post-processing. Hilar and mediastinal nodes were considered positive if the short axis was > 1 cm on chest CT images.

The inclusion criteria were as follows: (1) GGO lesions confirmed by high-resolution CT; (2) histopathologically confirmed adenocarcinoma; (3) systematic lymph node dissection that met the current standards (i.e. all lymph node stations 2–4 and 7–12 on the right side and stations 4–12 on the left side, according to the American Thoracic Society classification); and (4) tumor diameter ≤ 3 cm. Exclusion criteria were as follows: (1) preoperative distant metastasis with a history of lung cancer or new adjuvant chemotherapy; (2) cardiopulmonary function not suitable for segmentectomy or lobectomy; (3) patients with multiple lesions;and (4) no systematic lymph node dissection. There were 327 eligible patients.

Patients were divided into four groups according to the extent of the radiologic findings of GGO on preoperative chest CT. The consolidation/tumor ratio (C/T)% was defined as the proportion of the maximum consolidation diameter divided by the maximum tumor diameter. Four groups were defined as follows: PGGO, GGO-dominant type (C/T ≤ 0. 5), solid component dominant type (C/T > 0.5 to < 1), and solid tumor.

All patients underwent complete thoracoscopic surgery, with no transthoracic surgery or secondary surgery. For peripheral nodules, partial lobectomy (wedge resection or segmentectomy) was performed and the sample was then sent for frozen sectioning. If the frozen-pathology results indicated adenocarcinoma in situ or microinvasive adenocarcinoma, systemic lymph node dissection was performed, but in the case of invasive adenocarcinoma, lobectomy and systemic lymph node dissection were performed. Deep nodules were treated initially with segmentectomy or lobectomy, followed by systemic lymph node dissection. In our department, systematic lymph node dissection is required to include stations 2–4 and 7–12 in the right lung and 4–12 in the left lung.

### Statistical analysis

All the data were analyzed using SPSS 18.0 statistical software. Risk factors for lymph node metastasis were analyzed by *t*-tests, χ^2^ tests, and logistic regression. A value of *P* < 0.05 was considered significant.

## Results

Clinical data for the 327 patients are presented in Table 1. Twenty-six patients (7.95%) had mediastinal lymph node metastasis, including 10 (3.1%) with only N1 lymph node metastasis, five (1.5%) with only skip N2 metastatic lymph nodes with no N1 positive nodes, and 11 patients (3.4%) with both N1 and N2 lymph node metastasis. No mediastinal lymph node metastasis was present in the PGGO and GGO groups, while nine patients (9/120) with solid component dominant type and 17 (17/114) with pure solid tumor type had mediastinal lymph node metastasis.

**Table 1:** Clinicopathologic characteristics

Variable	Total	pN0	pN1 + N2	X2	*P* value
All patients	327	301	26		
Age (years)				2.264	0.322
≥ 60	172	162	10		
< 60	155	139	16		
Gender				5.663	0.017
Male	121	117	4		
Female	206	184	22		
Smoking history				1.593	0.207
Present	139	131	8		
Absent	188	170	18		
Tumor size (cm)				29.986	0.000
< 1.0	79	79	0		
1.1–2.0	140	135	5		
2.1–3.0	108	87	21		
Consolidation tumor ratio(C/T)				14.676	0.001
0	43	43	0		
0 to ≤0.5	50	50	0		
0.5to<1	120	111	9		
1	114	97	17		
CEA level (ng/mL)				5.246	0.022
≤ 5	268	251	17		
>5	59	50	9		
Tumor location				0.569	0.966
Right upper lobe	102	95	7		
Right middle lobe	31	28	3		
Right lower lobe	53	49	4		
Left upper lobe	79	73	6		
Left lower lobe	62	56	6		
Operations type				5.560	0.062
Wedge	29	29	0		
Segmentectomy	43	42	1		
Lobectomy	255	230	25		
Pathologic type				10.407	0.004
AIS	31	31	0		
MIA	52	52	0		
IA	244	218	26		
Lymphovascular invasion				53.189	0.000
Present	51	34	17		
Absent	276	267	9		
Pleural invasion				44.469	0.000
Present	46	31	15		
Absent	281	270	11		

*PGGO* pure ground-glass opacity, *AIS* adenocarcinoma in situ, *MIA* minimally invasive adenocarcinoma, *IA* invasive adenocarcinoma.

Univariate analysis showed that age, smoking history, tumor location, and operation type were not correlated with lymph node metastasis, but sex, tumor diameter, solid composition, plasma carcinoembryonic antigen (CEA) level, pathological type, vascular tumor thrombus, and pleural invasion were all related to lymph node metastasis.

Taking lymph node metastasis as a dependent variable with metastasis assignment as 1 and non-metastasis assignment as 0 (Table 2), we conducted multivariate unconditional binary logistic regression analysis (SLE = 0.05, SLS = 0.10) with age, sex, smoking history, tumor size, consolidation tumor ratio, CEA level, pathologic type, lymphovascular invasion, and pleural invasion as independent variables.

**Table 2:** Assignment description of influencing factors

Influencing factor	Assignment description
Gender	Man =1, Female = 2
Age (year)	< 60 = 1, ≥ 60 = 2
Smoking history	No = 0, YES = 1
Tumor size (cm)	< 1.0 = 1, 1.0–2.0 = 2, 2.1–3.0 = 3
Consolidation tumor ratio(C/T)	
PGGO	No = 0, YES = 1
GGO-predominant	No = 0, YES = 1
Solid-predominant	No = 0, YES = 1
Solid	No = 0, YES = 1
Serum CEA (ng/ml)	≤5 = 1, >5 = 2
Tumor location	
Right upper lobe	No = 0, YES = 1
Right middle lobe	No = 0, YES = 1
Right lower lobe	No = 0, YES = 1
Left upper lobe	No = 0, YES = 1
Left lower lobe	No = 0, YES = 1
Operations type	
Wedge	No = 0, YES = 1
Segmentectomy	No = 0, YES = 1
Lobectomy	No = 0, YES = 1
Pathological type	
AIS	No = 0, YES = 1
MIA	No = 0, YES = 1
IMA	No = 0, YES = 1
Visceral invasion	No = 0, YES = 1
Pleural invasion	No = 0, YES = 1

The results identified tumor diameter, proportion of solid components, plasma CEA level, pathological type, vascular tumor thrombus, and pleural invasion as risk factors for lymph node metastasis (Table 3).

**Table 3:** Independent predictors of lymph node metastasis by multivariate analysis

Variables	HR	95% CI	P value
Tumor size	1.503	1.155–1.955	0.002
Serum CEA	1.574	1.217–2.036	0.001
Pathological type	3.515	1.027–12.033	0.042
Visceral invasion	2.913	2.046–4.146	0.000
Pleural invasion	2.099	1.513–2.911	0.000

*HR* hazard ratio, *CI* confidence interval.

## Discussion

The TNM staging standard for lung cancer was revised by the International Association for Lung Cancer Research, with the new version reflecting the prognostic importance of tumor diameter. Previous studies found that tumor diameter was positively correlated with lymph node metastasis. A previous study in China [7] found mediastinal lymph node metastasis rates of 0, 10.5, and 29.7% in patients with peripheral lung cancers with diameters of 0–1, 1–2, and 2–3 cm, respectively (*P* = 0.003), while Okada et al. [8] reported that the rate of lymph node metastasis increased as the tumor size increased. The current study found similar results.

Previous studies found that the proportion of solid components in pulmonary nodules was related to lymph node metastasis. Matsuguma et al. [9] divided pulmonary nodules into five grades according to the proportion of solid components and showed that solid components were an independent risk factor for lymph node metastasis, i.e., the higher the proportion of solid components, the stronger the invasiveness of the lung cancer. Chen et al. [10] conducted a retrospective analysis of 867 patients with stage T1 lung cancer and divided them into three groups according to the proportion of solid components: I (pure GGO, *n* = 553), II (1–50%, *n* = 160), and III (50–79%, *n* = 154). Lymph node metastasis occurred in 25 cases, including no patients in group I, 11 in group II, and 14 in group III. The lymph node metastasis rates in the four groups in the current study were 0, 0, 7.5, and 14.9%, with an overall lymph node metastasis rate of 8.0% in the four groups. The total lymph node metastasis rates were similar to those found by Ding et al. [11] and Haruki et al. [12].

Lymphovascular invasion occurs when a small vein, artery, or lymphatic vessel wall in the tumor is damaged or there is a tumor thrombus in the lumen. The condition can be divided into vascular and lymphatic cancer thrombi, with a vascular tumor thrombus being a prerequisite for tumor invasion of the vascular system and lymph node metastasis. Lymphovascular invasion is believed to be a predictor of poor prognosis in patients with lung cancer. Tomoshi et al. showed that the 5-year overall survival rate of patients with vascular cancer thrombus was similar to that of stage IB patients without lymphovascular invasion [13]. It was therefore suggested that lymphovascular invasion should be considered as a factor in determining TNM stage. Furthermore, patients with phase IA lung cancer and lymphovascular invasion required postoperative oral uracil chemotherapy, which increased the 5-year survival rate from 66.6 to 93.3% [13].

Recent studies found that pathological type, pleural invasion, and CEA level were also important risk factors for lymph node metastasis [14, 15], as in the current study.

There is currently no clinical consensus on the need for segmental resection or lobectomy, and lymph node system cleaning or sampling in patients with early lung cancer. Both National Comprehensive Cancer Network and American College of Chest Physicians guidelines regard standard lobectomy and lymph node sampling/dissection as the standard procedures for early resection of non-small cell lung cancer [16, 17]. However, some studies suggested that there was no difference in long-term survival rates between patients with early lung cancer undergoing sublobectomy and lobectomy for tumors < 2 cm in diameter [18]. The American College of Chest Physicians guidelines suggest that sublobectomy can also be performed for pure GGOs with a diameter < 2 cm [17]. Tsutani et al. showed that, for GGO-dominant T1b tumors, 3-year recurrence-free survival rates were similar in patients who underwent lobectomy (93.7%), segmentectomy (92.9%), and wedge resection (100%) (*P* = 0.66) [19].

It is currently difficult to predict the existence of lymph node metastasis before surgery for early clinical lung cancer. Regarding intraoperative lymph node management, the benefit of systematic lymph node cleaning over lymph node sampling remains unclear. The randomized controlled ACOSOG Z0030 trial found no differences in local and distant recurrence rates and survival rates between the two groups; however, systematic lymph node dissection allowed more accurate staging [20]. Moon et al. analyzed 358 patients with non-small cell lung cancers ≤3 cm in diameter [21]. The postoperative pathology was adenocarcinoma in 129 patients with GGO-predominant findings, including only one case of lymph node metastasis, and there was no significant difference in 5-year recurrence rates between patients without lymph node dissection and those with lymph node dissection or sampling. The authors therefore suggested that no mediastinal lymph node dissection or sampling could reduce the operation risk and duration and thus improve the postoperative condition in lung cancer patients with GGO [21]. In the current study, no patients with PGGO or GGO-dominated lung cancer had lymph node metastasis, consistent with the results of Ye et al. [22]. Ye et al. also considered that most PGGO nodules were adenocarcinoma in situ or minimally invasive and lymph node metastasis was rare, suggesting that lymph node dissection should not be performed during surgery. Jiang et al. published a consensus on the diagnosis and treatment of early pulmonary adenocarcinoma with ground glass nodules at Shanghai Lung Hospital, which also concluded that there was no need for lymph node cleaning or sampling during surgery for in situ and microinvasive adenocarcinoma [23].

This study had some limitations. First, it was a retrospective study, and second, it was conducted at a single-center with a relatively small number of cases. Further multicenter randomized trials are therefore needed to verify these results.

## Conclusions

This study identified tumor diameter, solid component ratio, serum CEA level, pathological type, lymphovascular invasion, and pleural invasion as independent risk factors for lymph node metastasis in patients with clinical stage IA lung adenocarcinoma. Non-mediastinal lymph node metastasis is rare in patients with lung adenocarcinoma dominated by GGO component, and adenocarcinoma with PGGO may thus be treated by excision or lobectomy, with no need for mediastinal lymph node cleaning or sampling.

## Data Availability

All data generated or analyzed during this study are included in this published article.

## Abbreviations

GGO: Ground glass opacity
CEA: Plasma carcinoembryonic antigen
PGGO: Pure ground-glass opacity
AIS: Adenocarcinoma in situ
MIA: Minimally invasive
IA: Invasive adenocarcinoma
